# Differences in β-lactamase activity and carbapenem resistance among the *Bacillus cereus* group

**DOI:** 10.1128/aac.01302-25

**Published:** 2026-02-09

**Authors:** Yuji Nishihara, Ryuichi Nakano, Akiyo Nakano, Yuki Suzuki, Miho Ogawa, Ryuji Sakata, Hisakazu Yano, Kei Kasahara

**Affiliations:** 1 Department of Infectious Diseases, Nara Medical University 12967, Kashihara, Nara, Japan; 2 Department of Microbiology and Infectious Diseases, Nara Medical University 12967, Kashihara, Nara, Japan; 3 Department of Infectious Diseases, Japanese Red Cross Narita Hospital 13681, Narita, Chiba, Japan; 4 Department of Bacteriology, BML Inc., Kawagoe, Saitama, Japan; The Peter Doherty Institute for Infection and Immunity, Melbourne, Victoria, Australia

**Keywords:** enzyme activity, metallo-β-lactamase, carbapenemase, *Bacillus luti*, *Bacillus cereus* group

## Abstract

The *Bacillus cereus* group causes severe nosocomial infections. This group carries the chromosomal β-lactamases, including *bla1* and *BcII*, which contribute to β-lactam resistance; however, the β-lactam resistance mechanisms are poorly understood. We performed genomic and phenotypic analyses of 48 clinical isolates from blood cultures and the reference strain ATCC14579 to clarify these mechanisms. Genomic analyses included species identification, multilocus sequence typing (MLST), and detection of β-lactamase genes using whole-genome sequencing. β-Lactam susceptibility testing, enzyme activity assays, and RT-qPCR of β-lactamases were performed. For this analysis, we developed a method to measure the enzyme activity of *B. cereus* group. The 48 isolates comprised three species (30 *Bacillus mosaicus*, 9 *Bacillus cereus sensu stricto* (*s.s*.), and 9 *Bacillus luti*) and 28 sequence types. Although all *B. luti* isolates lacked carbapenemase genes, they exhibited higher minimum inhibitory concentration (MIC) ranges for ampicillin and meropenem. The enzyme activity patterns were categorized as constitutive, inducible, or silent. All *B. luti* isolates and some *B. mosaicus* and *B. cereus s.s*. isolates displayed constitutive enzyme activity for penicillin G, whereas most *B. mosaicus* and *B. cereus s.s*. isolates displayed inducible activity, and five displayed silent activity. In the inducible group, the induced activity appeared to be accompanied by elevated penicillinase and carbapenemase expression. This is the first study to demonstrate interspecies variability within the *B. cereus* group regarding the presence of carbapenemase genes and β-lactam resistance profiles. These findings provide crucial insights into β-lactam resistance mechanisms in this bacterial group and provide a foundation for further research.

## INTRODUCTION

The *Bacillus cereus* group comprises gram-positive, spore-forming rod bacteria that are ubiquitously distributed in natural environments (1, 2). This group includes closely related species with high genetic similarity, such as *Bacillus anthracis*, *Bacillus cereus*, and *Bacillus thuringiensis* (3). *B. anthracis*, harboring virulence plasmids pXO1 and/or pXO2, causes anthrax, which is potentially fatal. *B. cereus* mainly causes gastrointestinal infections such as food poisoning in humans but can also cause severe, invasive infections, particularly in immunocompromised individuals and neonates. *B. thuringiensis* causes lethal infections in insects and is widely used as a biopesticide (4). To date, approximately 20 species have been proposed for inclusion in the *B. cereus* group (3, 5). Specific species within the *B. cereus* group cannot be reliably differentiated using the phenotypic identification method or matrix-assisted laser-desorption/ionization time-of-flight mass spectrometry (MALDI-TOF MS) with commercially available databases (3, 6). Whole-genome sequencing (WGS) is required for accurate identification of species within this group (3, 7). Because of the difficulties with identification, detailed species classification is not possible in routine clinical laboratory practice.

Except for *B. anthracis*, most species within the *B. cereus* group are resistant to penicillins and cephalosporins (2). Penicillin susceptibility is frequently used to differentiate *B. anthracis* from other species of the *B. cereus* group, such as *B. cereus* and *B. thuringiensis* (8), but sporadic penicillin resistance of *B. anthracis* has been reported (9). In the *B. cereus* group, including *B. anthracis*, the expression of chromosomal β-lactamase genes is thought to be partially responsible for β-lactam resistance (8, 9). Chromosomally encoded β-lactamases include serine-β-lactamase, *bla1*, and metallo-β-lactamase, *bla2* (9, 10). In penicillin-sensitive *B. anthracis* strains, both *bla1* and *bla2* are almost silent transcriptionally, whereas semiquantitative RT-PCR analysis showed that these genes are expressed in penicillin-resistant strains (9, 11).

Previous studies have confirmed that *bla1* contributes to penicillin resistance. The deletion of *bla1* gene in penicillin-resistant *B. anthracis* strains results in a dramatic decrease in the minimum inhibitory concentration (MIC) of ampicillin (12), and *B. thuringiensis*, a species genetically similar to *B. anthracis*, also demonstrates a decreased MIC of ampicillin with the elimination of *bla1* (13). However, the implications of *bla2* for the susceptibility to carbapenems are not well understood. Discovered in 1966, *bla2* was the first extracellular metallo-β-lactamase to be discovered (14). Despite the presence of chromosomal carbapenemase, members of the *B. cereus* group are generally sensitive to carbapenem antibiotics (5, 15), and the production of *bla2* is insufficient to confer carbapenem resistance (9, 12).

The regulatory mechanisms underlying β-lactamase expression in the *B. cereus* group have been gradually elucidated, with recent studies demonstrating that extracytoplasmic function (ECF) sigma factors play a key role in this process (8, 13). However, the detailed regulatory mechanisms, especially those for carbapenemase expression, remain poorly understood.

In bacteria, the core RNA polymerase (RNAP) associates with a sigma factor to form the holoenzyme, which enables promoter recognition and transcription initiation. Sigma factors determine promoter specificity, thereby directing RNAP to transcribe distinct sets of genes (16). ECF sigma factors are a subfamily of σ^70^ sigma factors. They typically regulate cellular processes related to the cell envelope and are, therefore, termed “extracytoplasmic function” sigma factors (16). The general regulatory pathway of ECF sigma factors involves a membrane-bound anti-sigma factor that sequesters the sigma factor on the membrane, thereby suppressing its activity. Upon the detection of an extracytoplasmic signal, the anti-sigma factor is proteolytically degraded, releasing the sigma factor to activate transcription of the target genes (16, 17).

In the *B. cereus* group, SigP functions as an ECF sigma factor, and its activity is inhibited by its cognate anti-sigma factor, RsiP. These components are thought to participate in the regulation of β-lactamase expression. Penicillin-resistant *B. anthracis* strains constitutively express β-lactamase activity, whereas the deletion of the *sigP–rsiP* locus completely abolishes it (8). In *B. thuringiensis*, SigP regulates multiple β-lactamases and penicillin-binding proteins (PBPs), potentially contributing to β-lactam resistance (13). Additionally, ECF sigma factors upregulate β-lactamase activity in response to β-lactam antibiotic exposure (8, 13). However, few studies have investigated the levels of metallo-β-lactamase expression or the mechanisms of carbapenem resistance, and the mechanisms of metallo-β-lactamase and its contribution to carbapenem resistance remain unclear. Carbapenems are a crucial therapeutic option for treating infections caused by the *B. cereus* group (5); therefore, understanding carbapenem resistance mechanisms has important implications for treating clinical infectious diseases.

Measuring enzyme activity is crucial for clarifying the mechanisms of resistance to β-lactam antibiotics. However, in *Bacillus* species, β-lactamase activity has not been accurately quantified. Gram-positive bacteria secrete the β-lactamase enzymes that they produce into the extracellular environment (18, 19). Although previous studies have measured enzyme activity in liquid culture supernatants (11, 20), these measurements have not been normalized to protein content; therefore, enzyme activity assays have not been standardized.

In this study, we used 48 clinical isolates of the *B. cereus* group from patient blood cultures and conducted detailed species identification based on WGS analysis. We developed a novel method of quantifying enzyme activity toward β-lactam antibiotics and performed quantitative reverse transcription polymerase chain reaction (RT-qPCR) on a subset of the isolates to measure the levels of penicillinase and metallo-β-lactamase expression. This revealed that β-lactamase activity manifests in three distinct categories: constitutive, inducible, and silent and that carbapenem resistance levels and the expression of chromosomal β-lactamases vary considerably among species in the *B. cereus* group.

## RESULTS

### Species and MLST

**Fig 1:**
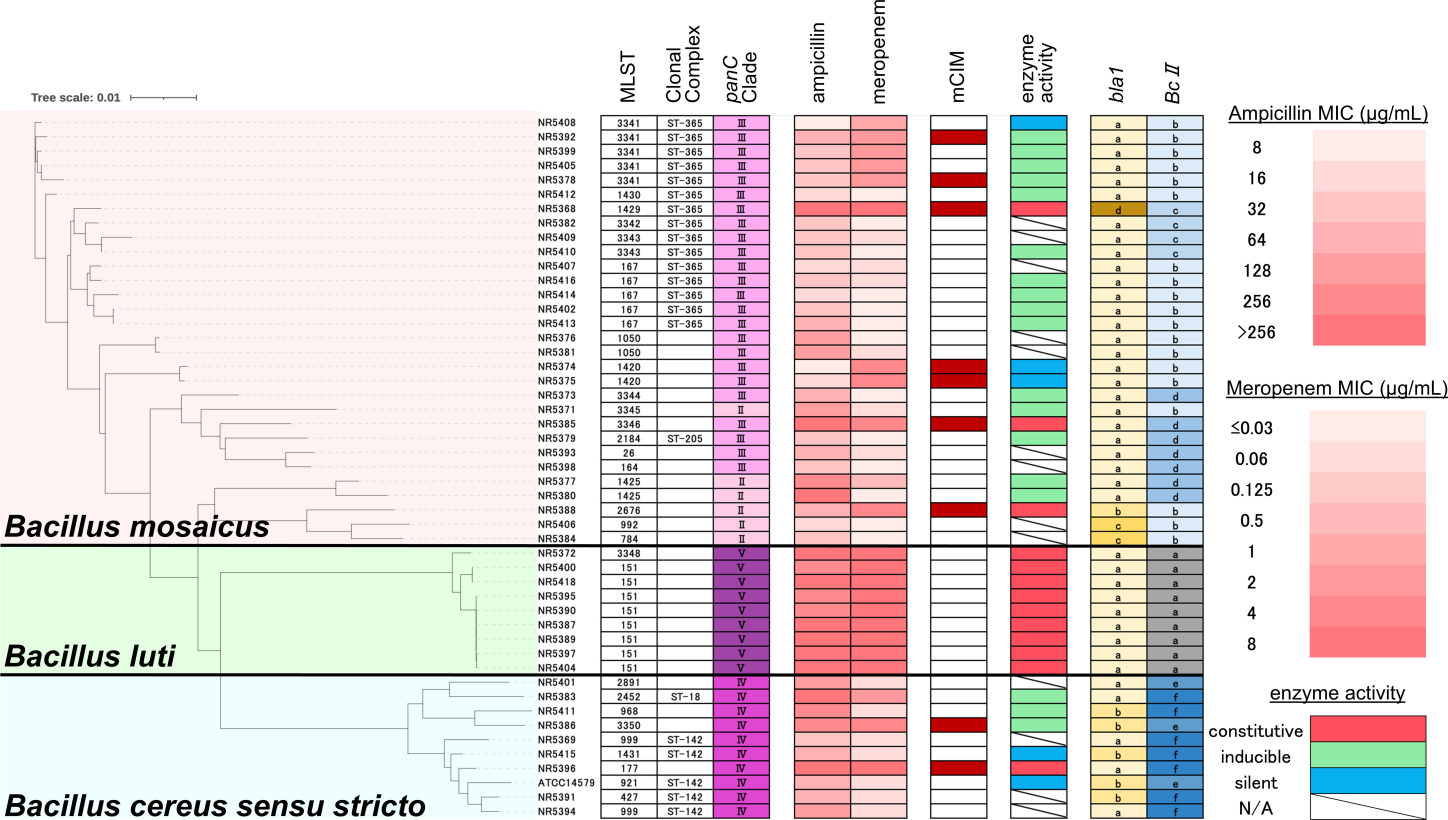
Phylogenetic tree of 48 clinical isolates from the *Bacillus cereus* group and the reference strain *B. cereus* ATCC 14579, with associated antimicrobial susceptibility, mCIM results, enzyme activity, and β-lactamase gene profiles. A phylogenetic tree of 48 NR isolates of *Bacillus cereus* group combined with the reference strain *B. cereus* ATCC 14579 was constructed using Mashtree (22). All isolates were classified into three species: *Bacillus mosaicus*, *Bacillus luti*, and *Bacillus cereus sensu stricto* (*s.s*.). A total of 28 distinct sequence types (STs) were identified. *B. cereus s.s*. isolates belonged to *panC* group IV, *B. luti* belonged to group V, and *B. mosaicus* belonged to groups II or III. Minimum inhibitory concentrations (MICs) of ampicillin and meropenem were represented using a color gradient from white (low MICs) to red (high MICs). Positive results in the mCIM are indicated by red boxes. The enzyme activity categories are shown with colored boxes; red indicates constitutive activity, green indicates inducible activity, and blue indicates a silent phenotype. The genetic structures around *bla1* and *BcII* are shown based on the classifications shown in Fig. 2 and 3, respectively. Abbreviation: mCIM, modified carbapenem inactivation method.

Using the average nucleotide identity (ANI) method of the BTyper3 system (21), 48 isolates were taxonomically classified as three different species: 30 *Bacillus mosaicus*, 9 *Bacillus cereus sensu stricto* (*s.s*.), and 9 *Bacillus luti* (Fig. 1). All *B. cereus s.s*. isolates were classified as *panC* group IV, and all *B. luti* isolates were classified as *panC* group V. The *B. mosaicus* were classified as either *panC* group II or III.

MLST analysis revealed that the 48 isolates were distributed across 28 distinct sequence types (STs). Of the nine *B. luti* isolates, eight were ST151. Of the 30 *B. mosaicus* isolates, 5 isolates each were ST167 and ST3341, and 2 isolates each were ST3343, ST1050, ST1420, and ST1425. Of the 30 *B. mosaicus* isolates analyzed, 15 were classified within clonal complex (CC) 365. The STs of *B. cereus s.s*. were different for all isolates, but four (44%) belonged to CC142. The species, MLST sequence type, CC, *panC* phylogenetic group, and other detailed genomic information are shown in Fig. 1 and Table S1.

### Antimicrobial susceptibility and the modified carbapenem inactivation method

The MICs (µg/mL) for ampicillin and meropenem, and the modified carbapenem inactivation method (mCIM) results, are shown in Fig. 1. The MICs of both antibiotics against *B. mosaicus* and *B. cereus s.s*. displayed a broad distribution. In contrast, *B. luti* exhibited consistently elevated MIC values for both β-lactams.

Notably, despite higher MICs for meropenem, all *B. luti* isolates tested negative using mCIM. Among the other two species, 7 (23%) *B. mosaicus* and 2 (22%) *B. cereus s.s*. isolates showed positive mCIM results. For these two species, all isolates with meropenem MICs >4 µg/mL were mCIM positive. Among the isolates, 4 *B. mosaicus* isolates and 1 *B. cereus s.s*. isolate had a meropenem MIC of 2 µg/mL, and 2 of the *B. mosaicus* isolates were mCIM positive. Importantly, all isolates with an MIC of ≤1 µg/mL were mCIM negative.

### Presence of β-lactamase genes among each species

**Fig 2:**
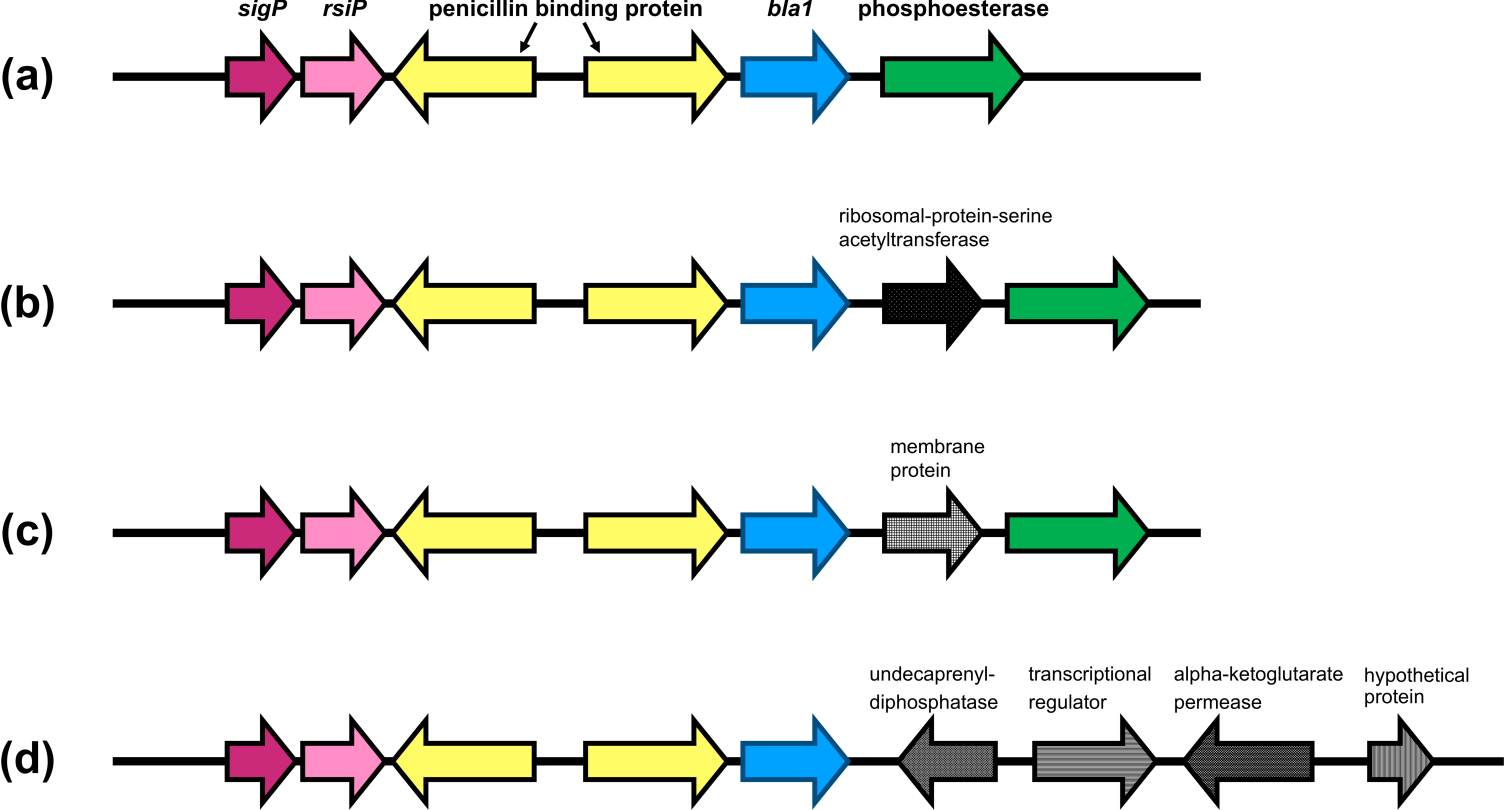
Schematic representation of the genetic context surrounding *bla1*. The upstream region of *bla1* was highly conserved across isolates and consistently contained *sigP*, *rsiP*, and two penicillin-binding protein genes. In contrast, the downstream region was classified into four distinct types based on the gene located immediately downstream of *bla1*. Forty of 48 isolates belonged to Group a; 5 isolates (NR5386, NR5388, NR5391, NR5411, and NR5415) and the reference strain, *B. cereus* ATCC 14579, belonged to Group b; two isolates (NR5384 and NR5406) belonged to Group c; and NR5368 belonged to Group d. Purple arrows represent *sigP*; pink, *rsiP*; yellow, penicillin-binding protein; blue, *bla1*; green, phosphoesterase; and black patterned arrows represent other genes. Detailed information on the isolates and their corresponding genetic types is provided in Table S2.

The *bla1* gene was found in all 48 isolates. The genetic structure surrounding *bla1* was highly conserved, with the sigma factor genes *sigP* and *rsiP* arranged sequentially, followed downstream by two types of PBPs and *bla1*. In contrast, the downstream region of *bla1* exhibited strain-specific variability, with diverse genetic elements, such as phosphoesterases, acetyltransferases, and membrane proteins, present in different isolates (Fig. 2).

The homology levels of *bla1* ranged from 85% to 99% at the nucleotide level and from 84% to 99% at the amino acid level. Relative to the ATCC reference strain, nucleotide homology varied among species. The homology ranged from 97% to 99% in *B. cereus s.s*., 91% to 92% in *B. luti*, and 85% to 99% in *B. mosaicus*.

The *BcII* (*bla2*) gene was detected in all the *B. cereus s.s*. and *B. mosaicus* isolates analyzed. In contrast with the relatively conserved region surrounding *bla1*, the genetic structure surrounding *BcII* displayed considerable variability. Notably, sigma factor genes such as *sigP* and *rsiP* were absent in the vicinity of *BcII*. Instead, the genetic region around *BcII*, and the corresponding genomic locus in *B. luti*, contained genes encoding lysozymes and hydroxylamine reductases, along with several genes of unknown function.

**Fig 3:**
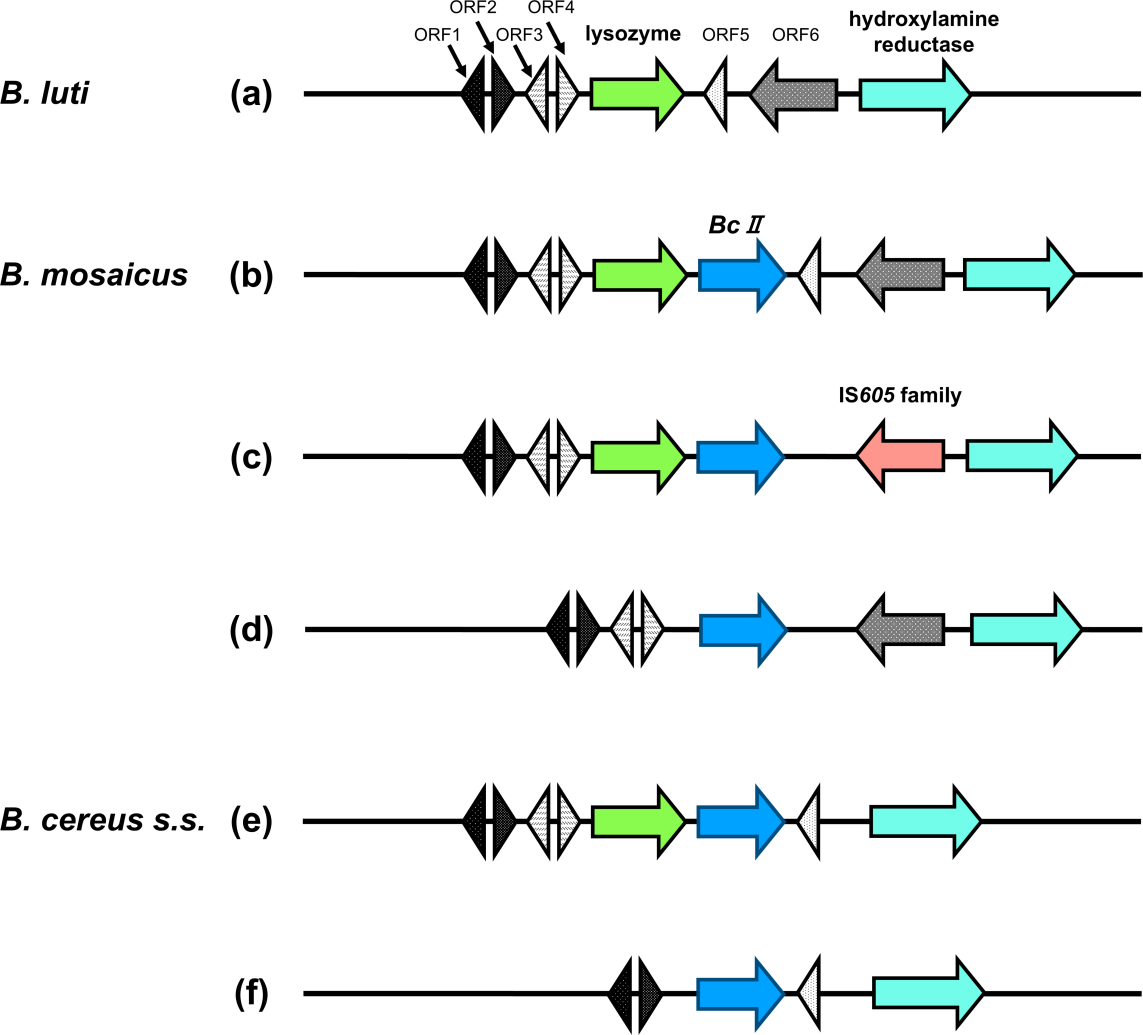
Schematic representation of the genetic context surrounding *BcII*. The genetic structure surrounding *BcII* varied between species. All nine isolates of *B. luti* completely lacked the *BcII* gene (type a). *B. mosaicus* isolates were classified into three distinct structural types (19 type b, 4 type c, and 7 type d) based on the presence or absence of lysozyme and IS*605* family elements. *B. cereus s.s*. was divided into two types (2 NR isolates and the reference strain *B. cereus* ATCC 14579 were type e, and 7 isolates were type f), depending on the presence of lysozyme. Green arrows represent lysozyme; blue, *BcII*; red, IS*605* family; light blue, hydroxylamine reductase; gray, uncharacterized protein; black with pattern, hypothetical protein (ORF1–ORF6). Detailed information on the isolates and their corresponding genetic types is provided in Table S2. Abbreviation: ORF, open reading frame.

In *B. mosaicus* and *B. cereus s.s*., *BcII* appeared to be inserted between the forward-oriented lysozyme gene and the reverse-oriented hypothetical protein found in *B. luti* (Fig. 3). The genetic structure of *B. luti* is shown in Fig. 3a, and the genetic structures of 19 of the 30 *B. mosaicus* are shown in Fig. 3b. In four *B. mosaicus* isolates (NR5368, NR5382, NR5409, and NR5410), an IS605 element was present downstream of *BcII* (Fig. 3c). Additionally, in seven *B. mosaicus* isolates (NR5373, NR5377, NR5379, 5380, NR5385, NR5393, and NR5398), the lysozyme gene immediately upstream of *BcII* was absent (Fig. 3d).

In *B. cereus s.s*., two isolates (NR5386 and NR5401) retained the lysozyme gene directly upstream of *BcII* (Fig. 3e). This lysozyme gene was absent in the remaining seven *B. cereus s.s*. isolates (NR5369, NR5383, NR5391, NR5394, NR5396, NR5411, and NR5415) (Fig. 3f). None of the bacterial isolates in this study showed evidence of genetic structures, such as homologous recombination or transposons, associated with the transmission of antimicrobial resistance genes.

The homology of *BcII* among the isolates ranged from 87% to 100% at the nucleotide level and from 86% to 100% at the amino acid level. The nucleotide homology of *BcII* was high in *B. cereus s.s*., ranging from 94% to 100%. In *B. mosaicus*, one isolate (NR5385) showed 99% homology, whereas the other isolates showed homology ranging from 87% to 89%. The detailed data on the homology and structure of each β-lactamase are summarized in Table S2.

### Enzyme activity

In this study, we developed a novel method for measuring the enzyme activity of β-lactamases per unit mass of protein in gram-positive bacteria. This method is based on the release of β-lactamases produced by gram-positive bacteria into the extracellular environment (18, 19). The technique involves centrifuging the supernatant of liquid culture media to isolate and quantify enzyme activity against β-lactam antibiotics. In addition to ATCC14579, 35 of the 48 clinical isolates from blood culture were chosen for enzyme activity analysis. The enzyme activity against penicillin G and meropenem was assessed in all 23 isolates, with meropenem MICs ≥0.5 µg/mL. For the remaining 25 isolates with meropenem MICs <0.5 µg/mL, a subset of 12 isolates was selected to ensure a balanced distribution of ampicillin MIC values.

Fig. 1 shows the phylogenetic tree of 49 (48 NR isolates and ATCC14579) *B. cereus* group isolates with the results of susceptibility testing, mCIM, enzyme activity, and β-lactamase genes.

#### Penicillin G

**Fig 4:**
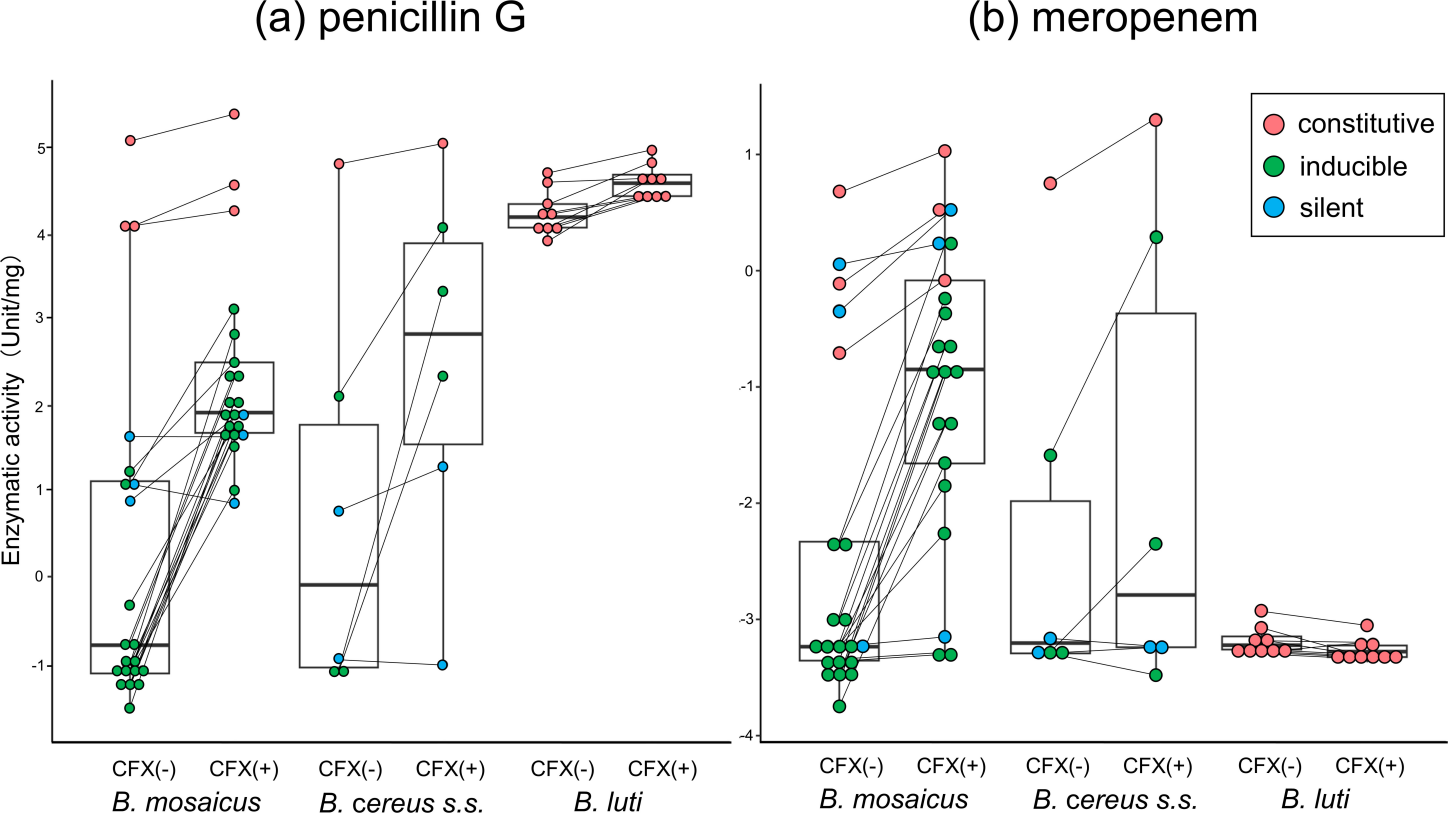
Enzyme activity against penicillin G (**a**) and meropenem (**b**). Boxplots were generated using the statistical software R version 4.3.2 (23) and ggplot2 (24) to show the distribution of the enzyme activity. The box represents the interquartile range (IQR), the horizontal line indicates the median, and the whiskers extend to 1.5 × IQR. Outliers are plotted as individual points. The enzyme activity was plotted for each bacterial isolate. Data points obtained with (+) and without (–) cefoxitin (CFX) induction are connected by a line to illustrate inducibility. The color of each dot represents the mode of penicillin G enzyme activity: red indicates the constitutive type, green the inducible type, and blue the silent type. The enzyme activity values (U/mg) are shown on a logarithmic scale. The β-lactamase activity values are the mean of three measures, with standard deviations within 10%.

The enzyme activity against penicillin G is shown in Fig. 4a. We identified three types of regulation system based on the relationship between enzyme activity levels and cefoxitin induction: constitutive, inducible, and silent. The constitutive group was defined as isolates with enzyme activity against penicillin G >50 U/mg without cefoxitin induction. The enzyme activity of the other two groups was <10 U/mg before induction. Isolates in which cefoxitin induction increased enzyme activity by more than threefold were classified as inducible, whereas isolates with a less than threefold increase were classified as silent.

In addition to three *B. mosaicus* isolates (NR5368, NR5385, and NR5388) and one *B. cereus s.s*. isolate (NR5396), all *B. luti* isolates were included in the constitutive group. Most *B. mosaicus* and *B. cereus s.s*. isolates were included in the inducible group, and three *B. mosaicus* isolates (NR5374, NR5375, and NR5408) and two of *B. cereus s.s*. isolates (NR5415 and ATCC14579) were included in the silent group.

#### Meropenem

The enzyme activity against meropenem is shown in Fig. 4b. Notably, all *B. luti* isolates had low activity and were not induced by cefoxitin, consistent with their lack of metallo-β-lactamase. The *B. mosaicus* and *B. cereus s.s*. isolates in the constitutive group also showed high enzyme activity against meropenem and penicillin G.

**Fig 5:**
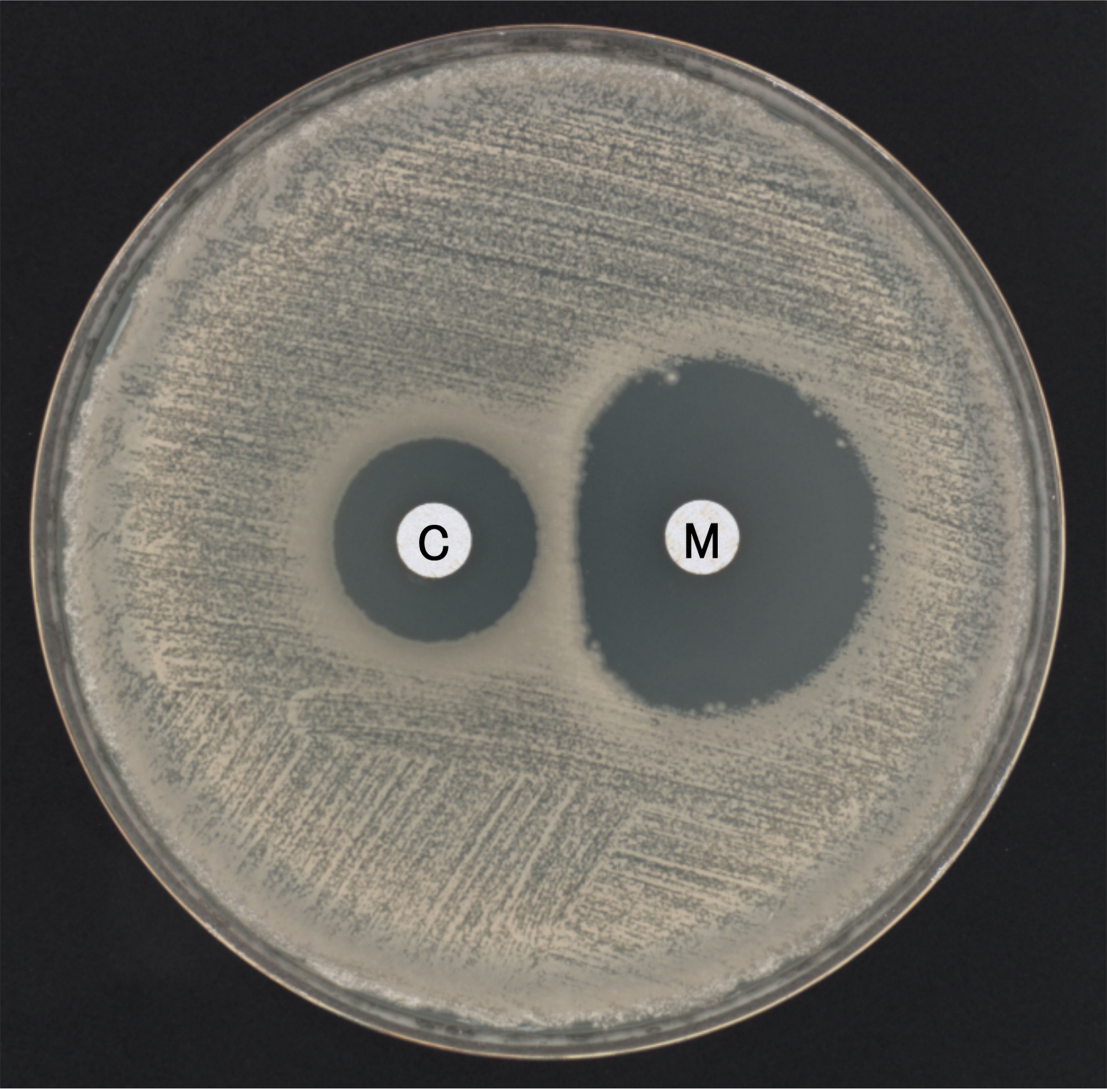
D-shaped zone of inhibition in inducible *B. mosaicus*. D-shaped zone of inhibition around the meropenem (M) disk adjacent to the cefoxitin (C) disk. Disks containing meropenem (10 µg) and cefoxitin (10 µg) were placed 20 mm apart on a Mueller-Hinton agar that had been inoculated overnight with *B. mosaicus* NR5416 in The induction group. The double-disk test was conducted in triplicate, and each replicate showed a similar D-shaped zone of inhibition.

In the inducible group, 13 of 18 isolates showed induced activity against meropenem and penicillin G. For the remaining five isolates (NR5373, NR5379, NR5383, NR5411, and NR5412) in the inducible group, meropenem enzyme activity was not induced by cefoxitin. Among the 13 isolates with induced β-lactamase activity against meropenem, isolate NR5416 was selected as a representative isolate and was used to assess whether the presence of cefoxitin affects meropenem resistance using the broth-microdilution and disk-diffusion methods. The MIC of meropenem was originally 0.06 µg/mL. In the presence of cefoxitin (1 µg/mL) (the same concentration used in the β-lactamase induction experiment), the MIC of meropenem increased to 1 µg/mL. Subsequently, a disk diffusion test was performed using 10-µg meropenem and 10-µg cefoxitin disks placed 20 mm apart on a Mueller-Hinton agar plate, followed by overnight incubation. After incubation, the inhibition zone around the meropenem disk adjacent to the cefoxitin disk was flattened, forming a characteristic D-shaped zone of inhibition (Fig. 5).

Three silent isolates (NR5408, NR5415, and ATCC14579) showed minimal activity against meropenem, but NR5374 and NR5375 showed high levels of enzyme activity against meropenem, comparable with those of the constitutive group.

The detailed data on enzyme activity, antimicrobial susceptibility, and mCIM are summarized in Table S3. Meropenem hydrolytic activity exceeded 0.09 U/mg in all mCIM-positive isolates, suggesting that mCIM accurately reflected the carbapenemase activity in *B. cereus* group isolates.

### RT-qPCR

In addition to the ATCC14579 strain, the expression levels of *bla1* and *BcII* were quantified in 23 of the 35 isolates with data available on enzyme activity using RT-qPCR. Representative isolates from each of the three enzyme activity groups were selected for RT-qPCR analysis.

The RT-qPCR results, β-lactam susceptibility profiles, mCIM outcomes, and enzyme activity measurements are summarized in Table 1. In the constitutive group, all isolates of *B. mosaicus* and *B. cereus s.s*. exhibited constitutive expression of both *bla1* and *BcII*, irrespective of cefoxitin induction. In contrast, all *B. luti* isolates demonstrated constitutive expression of *bla1* but showed no detectable expression of *BcII*. The fold change in β-lactamase gene expression following cefoxitin induction ranged from 0.8 to 1.6, indicating no significant change.

**TABLE 1:** Enzyme activity and category with results of MIC and mCIM^*a*^

Enzyme category	NR isolate	Species	MIC (µg/mL)		mCIM	Enzyme activity (unit/mg)^*b*^						mRNA^*c*^					
			AMP	MEM		PCNG	PCNG with CFX	Fold	MEM	MEM with CFX	Fold	bla1	*bla1* with CFX	Fold	BcII	*BcII* with CFX	Fold
Constitutive	5368	*B. mosaicus*	>256	8	Positive	60.29	72.76	1.21	1.97	2.80	1.42	0.62	0.86	1.39	8.22	9.08	1.11
	5385	*B. mosaicus*	>256	4	Positive	166.23	224.82	1.35	0.49	0.92	1.86	2.98	3.20	1.08	3.00	3.47	1.15
	5388	*B. mosaicus*	64	4	Positive	60.79	97.95	1.61	0.90	1.68	1.87	0.76	1.18	1.56	4.39	4.58	1.04
	5396	*B. cereus s.s*.	>256	8	Positive	125.31	159.57	1.27	2.13	3.67	1.73	0.76	0.85	1.13	2.57	2.30	0.90
	5372	*B. luti*	>256	8	Negative	113.46	148.25	1.31	0.04	0.03	0.95	1.82	1.55	0.85	<0.01	<0.01	<0.01
	5390	*B. luti*	256	8	Negative	67.47	88.17	1.31	0.04	0.04	0.94	1.91	1.58	0.83	<0.01	<0.01	<0.01
	5395	*B. luti*	256	8	Negative	61.70	101.64	1.65	0.04	0.04	1.04	1.88	1.68	0.89	<0.01	<0.01	<0.01
	5400	*B. luti*	256	8	Negative	50.84	110.93	2.18	0.05	0.04	0.76	1.40	1.70	1.22	<0.01	<0.01	<0.01
	5404	*B. luti*	>256	8	Negative	73.01	85.42	1.17	0.04	0.04	0.98	1.82	1.68	0.92	<0.01	<0.01	<0.01
Inducible	5386	*B. cereus s.s*.	256	4	Positive	8.30	60.01	7.23	0.20	1.34	6.55	0.28	1.63	5.86	<0.01	1.28	>100
	5402	*B. mosaicus*	32	≤0.03	Negative	0.27	5.18	19.20	0.03	0.41	13.87	0.10	2.52	26.47	<0.01	10.39	>100
	5405	*B. mosaicus*	32	2	Negative	0.72	5.62	7.83	0.04	0.54	13.69	1.09	28.89	26.58	<0.01	25.89	>100
	5413	*B. mosaicus*	64	≤0.03	Negative	0.30	5.96	20.08	0.03	0.42	13.05	0.50	2.86	5.78	3.76	22.19	5.90
	5416	*B. mosaicus*	32	0.06	Negative	0.45	10.16	22.68	0.05	0.79	16.07	0.43	4.45	10.27	<0.01	63.71	>100
	5371	*B. mosaicus*	128	0.06	Negative	0.29	6.69	22.83	0.03	0.26	7.99	2.44	2.63	1.08	0.35	11.06	31.36
	5380	*B. mosaicus*	>256	≤0.03	Negative	0.35	7.89	22.73	0.04	0.16	4.13	1.34	1.03	0.77	<0.01	0.87	>100
	5392	*B. mosaicus*	64	2	Positive	0.35	17.15	48.37	0.10	1.25	12.90	0.87	0.82	0.95	7.03	16.64	2.37
	5414	*B. mosaicus*	16	≤0.03	Negative	0.22	7.45	34.62	0.02	0.19	8.08	0.73	0.99	1.36	4.58	17.48	3.82
Silent	5374	*B. mosaicus*	8	4	Positive	5.19	5.39	1.04	1.06	1.27	1.21	1.16	1.14	0.99	1.69	1.07	0.63
	5375	*B. mosaicus*	16	4	Positive	2.43	6.49	2.68	0.71	1.70	2.39	0.91	1.10	1.21	0.91	0.49	0.54
	5408	*B. mosaicus*	8	1	Negative	2.81	2.35	0.84	0.04	0.04	1.12	2.37	2.14	0.90	0.93	<0.01	<0.01
	5415	*B. cereus s.s*.	64	0.06	Negative	0.38	0.36	0.93	0.04	0.04	0.93	1.40	1.21	0.86	1.38	0.93	0.67
	ATCC14579	*B. cereus s.s*.	64	0.06	Negative	2.15	3.61	1.68	0.04	0.04	1.01	1.00	0.89	0.89	1.00	0.51	0.51

^*a*^ Abbreviations: AMP, ampicillin; CFX, cefoxitin; MIC, minimum inhibitory concentration; mCIM, modified carbapenem inactivation method; MEM, meropenem; PCNG, penicillin G.

^*b*^ β-lactamase activities are the geometric means for three independent cultures. The standard deviations were within 10%.

^*c*^ Relative amount of *bla1 *or *BcII *mRNA compared to the ATCC 14579 basal level. All experiments were performed in triplicate. The standard deviations were within 10%.

The inducible isolates were categorized into two distinct groups based on the induction patterns of *bla1* and *BcII* expression. The first group exhibited induced expression of both *bla1* and *BcII*. This group included one *B. cereus s.s*. isolate (NR5386) and four *B. mosaicus* isolates (NR5402, NR5405, NR5413, and NR5416). In these isolates, cefoxitin induction led to a more than fivefold increase in *bla1* expression. Although *BcII* was predominantly silent under baseline conditions, its expression surged dramatically on induction. In contrast, the second inducible group consisted of four *B. mosaicus* isolates (NR5371, NR5380, NR5392, and NR5414) that demonstrated elevated *BcII* expression levels in response to cefoxitin, whereas *bla1* expression remained uninduced.

In the silent group, *bla1* and *BcII* were consistently expressed despite lower enzyme activity against penicillin G and were not induced by cefoxitin. Although NR5374 and NR5375 showed high levels of meropenem activity, their *BcII* expression levels (NR5374: 1.69, and NR5375: 0.91) were comparable to those of the three silent isolates with minimal meropenem activity (NR5408:0.93, NR5415:1.38, and ATCC 14579:1.00).

## DISCUSSION

This study provides a novel perspective on the distribution of species and genotypes within the *B. cereus* group isolated from clinical specimens. In addition, this is the first study to clarify the difference in β-lactam drug resistance among individual species within the *B. cereus* group and to identify the presence of three distinct types of regulatory mechanisms for β-lactamase activity: constitutive, inducible, and silent.

### Genomic analysis

The 48 isolates used in this study were classified into 28 different STs. Previous studies have demonstrated genetic diversity within the *B. cereus* group. For example, in studies conducted in the United States, an analysis of 85 environmental isolates identified 62 unique STs (25), and 55 clinical isolates were classified into 38 distinct STs (26). However, the genomic accumulation of specific STs has also been observed. For example, ST1420 is a predominant ST among *B. cereus* group responsible for recent nosocomial infections in Japan (27). A comprehensive genomic analysis of 191 *B. cereus s.s*. isolates from the National Center for Biotechnology Information (NCBI) database further revealed that clonal complex (CC) 142 is the most widespread genetic cluster within the global *B. cereus* group (28).

In our study, eight new STs (ST3341, ST3342, ST3343, ST3344, ST3345, ST3346, ST3348, and ST3350) were submitted to the online MSLT database (29). ST151, belonging to *B. luti*, was the most frequently identified ST; however, detailed information regarding the source of isolation or geographic distribution is missing in most previous studies. CC142 was the most prevalent cluster among *B. cereus s.s*., consistent with previous reports (28), and CC365 was the most frequently detected genetic cluster among *B. mosaicus*. As this study is based on data from a single center in Japan, the findings cannot be directly generalized to other hospitals or regions. Nevertheless, these results suggest the involvement of specific species or STs in human infections.

The genomic analysis revealed a distinct distribution of metallo-β-lactamase among the species. All *B. mosaicus* and *B. cereus s.s*. isolates carried *BcII*, whereas all *B. luti* isolates lacked this gene. This complete segregation suggests species-specific differences in *BcII* distribution.

Fifteen whole-genome assemblies of *B. luti* have been entered into the NCBI database to date, of which none contain the *BcII* gene (30). A study conducted in Italy analyzed 17 blood culture isolates, all of which were closely related to either *B. cereus s.s*. or *B. thuringiensis* and carried the *BcII* gene (31). Another study found that 76 of 85 environmental isolates (89%) harbored the *BcII* gene. Among the nine isolates lacking *BcII*, five were identified as *Bacillus pseudomycoides*, three as *B. cereus s.s*., and one as *B. mosaicus* (25). Integrating our findings with those of previous reports suggests that *B. luti* does not inherently possess *BcII*, whereas the majority of other species within the *B. cereus* group probably carry *BcII*.

Considering the genetic characteristics identified in this study, specifically, the species-specific variations in the surrounding structure of *BcII* suggest that the development of a refined strategy for species differentiation may be possible using methods such as PCR-based assays. Currently, accurate identification of the *B. cereus* group requires WGS. The development of a simplified species identification method would greatly enhance the efficiency of rapid microbial classification. We plan to conduct further research focusing on expanding the strain collection, refining the data set, and advancing the development of a rapid detection method.

### MIC distribution and mCIM

*B. cereus* group is inherently resistant to penicillins and cephalosporin β-lactam antibiotics due to the presence of chromosomally encoded β-lactamases (9, 32). Carbapenems are essential therapeutic agents for treating infections caused by this species (5); however, limited data are available on carbapenem susceptibility among species within the *B. cereus* group.

To our knowledge, this study is the first to demonstrate that the carbapenem susceptibility rate and mechanisms of carbapenem resistance vary among species within the *B. cereus* group and that the presence of *BcII* alone is not a reliable indicator of carbapenem resistance.

Notably, in this study, the *B. luti* isolates exhibited the highest meropenem MICs among the three species despite the absence of the *BcII* gene. All *B. luti* isolates tested mCIM negative, consistent with the absence of detectable carbapenemase activity. These findings suggest that carbapenem resistance in *B. luti* is mediated by mechanisms other than carbapenemase production.

To date, no comprehensive studies have been conducted focusing on non-β-lactamase-mediated mechanisms of carbapenem resistance in the *B. cereus* group. Generally, modified PBPs associated with β-lactam resistance are more common in gram-positive bacteria than in gram-negative bacteria (33). Gram-positive bacteria, including *Streptococci pneumoniae*, *Streptococcus mitis/oralis*, and *Enterococcus faecium*, are resistant to carbapenems because of the reduced affinity of PBPs for these antibiotics (34–36). Alterations in PBPs are responsible for penicillin resistance in *Bacillus subtilis*, a species closely related to *B. cereus* group (37). Efflux pumps have been implicated in the resistance of certain isolates within the *B. cereus* group to β-lactam antibiotics, including ampicillin and cefuroxime (38). Although this remains a subject for future research, it is possible that *B. luti*-specific mechanisms, such as mutations in PBPs or the involvement of active efflux pumps, may contribute to carbapenem resistance. Further studies, including a larger collection of clinical isolates, are needed to clarify the species-specific mechanisms of carbapenem resistance within the *B. cereus* group.

The other two species, *B. mosaicus* and *B. cereus s.s*., all had *BcII* and displayed a wide range of MICs for meropenem. The isolates with higher MICs for meropenem were mCIM positive, which suggests that carbapenem resistance might be caused by carbapenemase production in these two species. The mCIM was originally developed to detect carbapenemase production in *Enterobacteriaceae* and *Pseudomonas aeruginosa* (39, 40), and its applicability to other bacterial species remains unclear. However, our study suggests that in the *B. cereus* group, mCIM may be a useful tool for predicting carbapenemase production in isolates with elevated meropenem MICs. In carbapenem-resistant isolates, this approach could be used to differentiate *B. luti*, which lacks *BcII*, from other species that harbor metallo-β-lactamase.

### Enzyme category and RT-qPCR

In this study, the β-lactamase activity in the *B. cereus* group was successfully measured using a newly developed method. The enzyme activity was able to be accurately assessed by concentrating the crude enzyme extract. Without concentration, the activity values could not be reliably determined, probably due to the low protein concentration (data not shown). In gram-negative bacilli, enzyme activity is typically assessed following sonication; however, in the *B. cereus* group, sonication did not yield a significant difference compared with that of non-sonicated samples (data not shown). This phenomenon may be attributable to the characteristic secretion of β-lactamase into the extracellular environment in gram-positive bacteria (18, 19). A positive correlation was observed between enzyme activity and β-lactamase gene expression levels in individual inducible isolates before and after cefoxitin induction, which suggests that the enzyme activity assay developed in this study is a reliable method for quantifying β-lactamase activity. Further research is warranted to evaluate the applicability of this assay to other gram-positive bacterial species.

This is the first study to identify three distinct types of β-lactamase activity in species within the *B. cereus* group: constitutive, inducible, and silent. In the constitutive group, all isolates of *B. luti* and several isolates of *B. mosaicus* and *B. cereus s.s*. showed the high enzyme activity for penicillin G without cefoxitin induction, which suggested this group constitutively produced β-lactamases. In accordance with the enzyme activity results, the β-lactamase gene was constitutively expressed in this group. In the silent group, all except two isolates of *B. mosaicus* and *B. cereus s.s*. demonstrated consistently low enzyme activity, and activity was not induced by cefoxitin exposure.

In the inducible group, baseline β-lactamase activity against penicillin G and meropenem was almost undetectable under baseline conditions, but cefoxitin dramatically induced this activity and upregulated transcription of *bla1* and *BcII*. In NR5416, the meropenem MIC increased from 0.06 µg/mL to 1.0 µg/mL in the presence of cefoxitin, and the meropenem disk produced a D-shaped inhibition zone when placed adjacent to a cefoxitin disk. Similar phenomena have been reported in Enterobacterales, where double-disk synergy tests have shown that certain β-lactam agents can induce β-lactamase expression, resulting in flattening of the inhibition zone surrounding a nearby β-lactam disk (41, 42). The double-disk result observed with NR5416 provides visual evidence of the capacity for metallo-β-lactamase induction.

The tripartite pattern of enzyme activity observed in this study might have arisen from the involvement of an ECF sigma factor. The β-lactamase activity of the *B. cereus* group is regulated by ECF sigma factors, including SigP and its associated anti-sigma factor, RsiP. Degradation of RsiP triggers SigP activation, which subsequently drives the transcription of *bla* genes (8). β-Lactam antibiotics such as cefoxitin have been shown to promote RsiP degradation and SigP activation, resulting in increased β-lactamase expression (43). Previous studies have reported that mutations in *rsiP* can affect β-lactamase activity. In one study, silico substitution of Valine82 or Serine84 of *rsiP* with tryptophan eliminated the predicted cleavage site and prevented SigP activation, whereas substitution of Serine84 with alanine markedly increased the predicted probability of signal-peptidase cleavage, resulting in constitutive SigP activity (44). Another study found that in a penicillin-resistant *B. anthracis* isolate, RsiP was truncated due to a genomic mutation that prevented it from sequestering the cognate SigP. Consequently, SigP remained continuously activated, leading to constitutive expression of *bla* genes (8). These previous findings suggest that the SigP/RsiP regulatory system plays a pivotal role in modulating the enzyme activity of β-lactamases.

In our study, all isolates contained homologs corresponding to *sigP/rsiP*. However, no specific mutations were identified that could differentiate between constitutive, inducible, or silent β-lactamase activity phenotypes. Specifically, no isolate harbored mutations at Valine82 or Serine84, and none of the isolates in the constitutive group carried the *rsiP* mutation responsible for truncated RsiP. Genomic analysis of *Bacillus anthracis* has shown that mutations in the *sigP-bla1* region cannot be exclusively used to predict penicillin resistance (11). A more comprehensive genomic analysis of sigma factors and other related elements in a larger sample of isolates could contribute to the elucidation of the regulatory mechanisms underlying β-lactamase production.

Another notable finding from the enzyme activity assay and RT-qPCR analysis is the lack of a consistent correlation between β-lactamase gene expression and enzyme activity. Despite the expression of *BcII*, isolates NR5392, NR5413, and NR5414 from the inducible group, and NR5408 and NR5415 from the silent group exhibited very low hydrolytic activity against meropenem. These findings indicate that *BcII* expression does not necessarily confer resistance to carbapenems. This observation is consistent with a previous report describing a penicillin-resistant *B. anthracis* strain, designated UT223, which expressed *BcII* but remained susceptible to meropenem, with a MIC of 0.06 µg/mL (12). Previous studies have demonstrated that specific amino acid substitutions within *BcII* can alter its enzyme activity toward β-lactam antibiotics. For example, individual substitutions such as G262S or N70S are associated with reduced catalytic efficiency against carbapenems, whereas their simultaneous presence results in enhanced catalytic activity against these agents (45). Although none of the isolates in this study harbored these specific mutations, the *BcII* gene sequences of our isolates showed a wide range of homology, with sequence similarities ranging from 87% to 100%, suggesting potential involvement of other as yet unidentified mutations that may influence catalytic efficacy and substrate specificity.

### Limitations

This study has several limitations. First, the analysis was based on a relatively small number of isolates obtained from a single institution. Although *B. luti* isolates identified in this study demonstrated elevated MICs for carbapenems and lacked the *BcII* gene, further validation using a more extensive and geographically varied isolate set is necessary to ascertain whether these features are species-specific.

Second, the mechanisms responsible for carbapenem resistance in *B. luti* have not yet been elucidated. In addition, among non-*B*. *luti* species, some isolates, such as NR5405 and NR5408, showed high MICs for meropenem despite exhibiting very low metallo-β-lactamase activity. This phenotypic characteristic is similar to that observed in *B. luti*, suggesting that *B. luti* and certain isolates of non-*B*. *luti* species may possess an unknown resistance mechanism.

Third, the regulatory systems of β-lactamase gene expression and the consequent effects on both enzyme function and antimicrobial susceptibility have not yet been well characterized. The observation that certain isolates express *BcII* while displaying minimal enzyme activity raises the possibility that gene expression alone may not lead to phenotypic resistance. This highlights the need for further investigation into regulatory pathways, genomic mutation, and variety, as well as the structural determinants of substrate specificity.

To address these limitations, future studies should incorporate a broader range of clinical isolates and employ integrated genomic, transcriptomic, and functional approaches. Such efforts will be instrumental in advancing our understanding of the molecular basis of β-lactam resistance and may ultimately inform the development of more effective diagnostic tools and therapeutic strategies.

### Conclusion

In this study, a comprehensive whole-genome analysis provides new insights into the distribution patterns of bacterial species and genotypes of *B. cereus* group isolated from blood cultures. Although the *B. cereus* group is generally recognized to harbor chromosomally encoded metallo-β-lactamases, *B. luti* lacked *BcII* yet exhibited high MICs for carbapenems. In addition, by developing a novel method for measuring enzyme activity, we identified three distinct types of activity: constitutive, inducible, and silent. Expanding this approach to additional bacterial species and isolates could lead to further facilitation of the understanding of β-lactam resistance.

## MATERIALS AND METHODS

### Bacterial isolates

A total of 48 isolates were collected from the blood cultures of different 48 patients between January 2009 and October 2021 at Nara Medical University Hospital, a tertiary teaching hospital in Japan with 992 beds. All isolates had already been identified as *B. cereus* group using MALDI-TOF MS of Vitek MS system (bioMérieux, France). Additionally, we included ATCC14579, a type strain of *B. cereus*, in our investigation.

### WGS and genomic analysis

For WGS analysis, genomic DNA was extracted using a QIAGEN Genomic-tip 500/G (Qiagen, Germany) and sequenced using MiSeq (Illumina, United States). The assembled sequences were annotated using DFAST with default parameters (46). *B. cereus* isolates were taxonomically classified using BTyper3 based on pairwise genomic similarity, which was calculated using an integrated method, average nucleotide identity based on BLAST (ANIBlast) (21). BTyper3 was used to perform the analysis of the *panC* gene phylogenetic group. Mashtree was used to cluster the genomes into a phylogenetic tree (22), which was visualized using iTOL v7.0 (47, 48). The presence of β-lactamase genes was determined using ABRicate v1.0.1 (49) based on the NCBI database (50). For β-lactamase genes in the *B. cereus* group, we adopted the designation “*bla1*” for penicillinase, and “*BcII*” for metallo-β-lactamase, according to the terminology used in the NCBI database.

### Antimicrobial susceptibility and modified carbapenem inactivation method

Based on the Clinical and Laboratory Standards Institute (CLSI) M45:ED3 guidelines, broth microdilution tests were conducted on every isolate to determine the MIC for ampicillin and meropenem (51). The modified carbapenem inactivation method (mCIM) was performed using a 10-µL loop for the detection of carbapenemase production (52). Each isolate was tested by mCIM in duplicate, and the interpretation of the results was concordant in both experiments.

### Enzyme activity

β-Lactamase activity was measured using a colorimetric assay (53) based on a modification of a previously described method (54). Bacterial strains were inoculated into Mueller-Hinton broth and incubated for 5 h with shaking at 37℃. For the induction assay, bacterial strains were cultured in Mueller-Hinton broth to mid-logarithmic phase and subjected to cefoxitin 1 μg/mL as the inducer for 3 h. After incubation for 5 h, the culture broth was centrifuged at 3,500 rpm for 20 min at 4℃. The supernatant was ultrafiltered using a Vivaspin Turbo 15 (Sartorius, Germany) with a 10,000 Da molecular weight cutoff filter. Filtrated supernatants (50 µL) were suspended in 2 mL of 50 mM phosphate buffers (pH 7.0) with 50 µM MgSO_4_. Enzyme activity was determined at 30℃ using a spectrophotometer (UV-1900i Plus; Shimadzu, Japan) with penicillin G (233 nm) and meropenem (298 nm) as the substrate. The final concentration of penicillin G and meropenem was adjusted to 100 µM. The enzyme activity was measured three times, and the mean value was used. Protein concentrations were measured using the Bradford assay (55). One unit of β-lactamase activity is equivalent to the amount of β-lactamase that hydrolyzes 1 μmol of β-lactams in 1 min at 30℃.

### RT-qPCR

The expression levels of *bla1* and *BcII* were measured using RT-qPCR. After incubation for 5 h, RNA was extracted from the cell pellet using an RNeasy Mini Kit (Qiagen, Germany) following the manufacturer’s protocol. Real-time PCR amplification was performed using the Power SYBR Green RNA-to-C_T_ 1-Step kit on a QuantStudio 5 Real-Time PCR system (Thermo Fisher Scientific, the United States). One-step real-time PCR was performed under the following conditions: 48°C for 30 min for cDNA synthesis, 95°C for 10 min for transcriptase inactivation, followed by 40 cycles of 95°C for 15 s and 60°C for 1 min. Based on a previous study, *gatB_Yqey* and *udp* were used as internal reference genes (56). The primers for *bla1* and *BcII* were newly designed in this study. To accommodate sequence variations, two reverse primers targeting *BcII* were combined in equal proportions. The relative expression levels of *bla1* and *BcII* were determined by 2^−ΔΔCt^ method, using the ATCC14579 strain without cefoxitin induction as a control sample. The primers used in this study are shown in Table 2. Real-time PCR assays were performed in triplicate for each sample, and the mean Ct value was used in the analysis.

**TABLE 2:** Primers used for the RT-qPCR assays in this study

Gene	Orientation	Primer sequence (5' to 3')	Product size (bp)	Reference
*bla1*	F	GCTATTCCAGGAGAYATTCG	161	This study
	R	ACRCCTGCACGAATAAGTTT		
*BcII*	F	AAGCAGTTCCTTCGAACG	99	This study
	R1	TCTCTATTAATTCCTTCGTTAAC		
	R2	TTTCTATTAGTTCCTTCGTTAAT		
*gatB_Yqey*	F	AGCTGGTCGTGAAGACCTTG	175	(56)
	R	CGGCATAACAGCAGTCATCA		
*udp*	F	ACTAGAGAAACTTGGAAATGATCG	101	(56)
	R	GACGCTTAATTGCACGGAAC		

## Data Availability

All sequence data have been deposited at DNA Data Bank of Japan (DDBJ) under BioProject accession number PRJDB17557. Accession numbers for the Illumina sequence data are listed in Table S1.

## SUPPLEMENTAL MATERIAL

Supplemental material aac.01302-25-s0001.xlsx Tables S1 to S3.

ASM does not own the copyrights to Supplemental Material that may be linked to, or accessed through, an article. The authors have granted ASM a non-exclusive, world-wide license to publish the Supplemental Material files. Please contact the corresponding author directly for reuse.
